# Serum Concentrations of Vascular Endothelial Growth Factor in Polish Patients with Systemic Lupus Erythematosus Are Associated with Cardiovascular Risk and Autoantibody Profiles

**DOI:** 10.3390/jcm14145133

**Published:** 2025-07-19

**Authors:** Katarzyna Fischer, Hanna Przepiera-Będzak, Marcin Sawicki, Maciej Brzosko, Marek Brzosko

**Affiliations:** 1 Independent Laboratory of Rheumatic Diagnostics, Pomeranian Medical University in Szczecin, Unii Lubelskiej 1, 71-252 Szczecin, Poland; maciej.brzosko@pum.edu.pl; 2 Department of Internal Diseases, Rheumatology, Diabetology, Geriatrics and Clinical Immunology, Pomeranian Medical University in Szczecin, Unii Lubelskiej 1, 71-252 Szczecin, Poland; hanna.przepiera.bedzak@pum.edu.pl (H.P.-B.); marek.brzosko@pum.edu.pl (M.B.); 3 Department of Imaging Diagnostics and Interventional Radiology, Pomeranian Medical University in Szczecin, Unii Lubelskiej 1, 71-252 Szczecin, Poland; marcin.sawicki@pum.edu.pl

**Keywords:** vascular endothelial growth factor, systemic lupus erythematosus, antiphospholipid antibodies, anti-endothelial cell antibodies, anti-double stranded DNA antibodies, anti-oxidized low-density lipoprotein antibodies, atherosclerosis, cardiovascular disease

## Abstract

**Background/Objectives**: This study was conducted to analyze the associations between vascular endothelial growth factor (VEGF) serum concentrations and immunological biomarkers, inflammatory parameters, classical atherosclerosis risk factors, and cardiovascular manifestations in systemic lupus erythematosus (SLE) patients. **Methods**: The project included 83 individuals suffering from SLE, with 20 healthy individuals as controls. The serum levels of VEGF were determined through the ELISA method using R&D Systems tests. Laboratory markers, autoantibody profiles, traditional atherosclerotic risk factors, and organ manifestations were evaluated. Atherosclerotic changes were determined based on several indices including carotid intima-media thickness, ankle-brachial index and high resistance index assessments. **Results**: The reference range of serum VEGF concentrations was established based on the 25th and 75th percentiles obtained in the controls. High VEGF levels were significantly correlated with the presence of selected anti-phospholipid antibodies such as anti-prothrombin (OR = 10.7; 95%CI: 2.1–53.4) and anti-beta2 glycoprotein I (OR = 3.5; 95%CI: 1.1–10.8), as well as cardiac disorders (OR = 8.0; 95%CI: 1.6–39.5). On the other hand, low concentrations of VEGF were significantly related to lower frequencies of anti-double-stranded DNA antibodies (OR = 0.31; 95%CI: 0.11–0.91) and anti-endothelial cell antibodies (OR = 0.30; 95%CI: 0.11–0.85). Patients with low VEGF levels showed significantly reduced risks of atherosclerotic lesions (OR = 0.24; 95%CI: 0.04–0.99) and vasculitis development (OR = 0.17; 95%CI = 0.03–0.91). **Conclusions**: In conclusion, VEGF’s pathogenetic role in SLE and SLE-related atherothrombosis is manifested in close correlation with aPLs which may enhance their direct impact on endothelium. High VEGF levels are helpful for identifying cardiovascular risk in patients, while low concentrations indicate lower disease activity, as well as a lower risk of organ involvement.

## 1. Introduction

Systemic lupus erythematosus (SLE) is a complex, chronic autoimmune disease characterized by multiorgan involvement and the production of a wide profile of autoantibodies [1]. The clinical heterogeneity of SLE is an effect of interactions among various etiopathogenetic factors, including genetic susceptibility, epigenetic and environmental factors, infections, and sex hormones [2]. SLE patients are prone to developing accelerated and premature atherosclerosis. Cardiovascular disease (CVD) is still one of the main causes of morbidity and mortality in this group [3]. The pathogenesis of accelerated atherosclerosis in SLE is not fully understood but there is a growing bulk of evidence that it relates to complex interactions among inflammation, classical risk factors, endothelial dysfunction, immune dysregulation, and therapeutics [4].

Vascular endothelial growth factor (VEGF) was first described by Senger et al. in 1983 [5]. VEGF is considered to be a potent angiogenetic factor that regulates many steps of the angiogenesis process, including vasodilatation, the permeability of the endothelial barrier, structural remodeling of the perivascular matrix, and the proliferation and relocation of endothelial cells [6]. It is produced by macrophages, fibroblasts, endothelial cells, and smooth muscle cells [7], and it contributes to many physiological and pathological processes such as embryonic development, wound healing, placenta formation, ocular neovascularization, tumor progression, endometriosis, and CVD [8]. It also contributes to the pathogenesis of autoimmune diseases, including rheumatoid arthritis [9], antiphospholipid syndrome (APS) [10,11], spondyloarthropathies (SpAs) [12,13,14,15,16], and SLE [17,18,19,20,21,22,23,24,25,26,27], including that with pediatric onset [28,29].

The current study aimed to analyze the complex relationships between VEGF serum levels and atherosclerosis, as measured through various imaging methods, atherosclerotic risk factors, cardiovascular manifestations, other organ involvement, and autoantibody profiles in lupus patients.

## 2. Materials and Methods

### 2.1. Study Participants

The bioethics committee at Pomeranian Medical University in Szczecin approved the study protocol (KB-0012/11/13), and we received written informed consent from all individuals participating in this research.

This cross-sectional original research was conducted in 83 Caucasian SLE patients who qualified for the study during routine visits to the outpatient clinic in the period from 2017 to 2018. The mean age of the patients was 44.8 years. The diagnosis was established in accordance with the American College of Rheumatology Classification criteria [30] and the 2012 Systemic Lupus International Collaborating Clinics (SLICC) criteria [31]. The median duration of the disease was 8.0 years. The Systemic Lupus Erythematosus Activity Index (SLEDAI) was used to assess SLE activity [32]. APS was diagnosed on the basis of criteria published in 2006 [33]. Additionally, other organ manifestations were included: renal involvement, Raynaud’s phenomenon, vasculitis, and cardiovascular disorders (coronary artery disease [CAD] and/or myocardial infarction, cerebrovascular disorders like cerebral stroke and/or transient ischemic attacks, left-ventricular function abnormalities, hypokinesis, relaxation abnormalities). We also collected data on the treatment schemes.

The control group comprised 20 healthy individuals who were carefully selected to be consistent with the patient group in terms of age and gender. No one had a confirmed history of CVD. Only one person was diagnosed with hypertension; no one was diabetic. All inflammatory markers were within the reference values. The control group’s lipid profiles, body mass index (BMI) values, smoking habits, oral contraceptive use, and family history of CVD did not differ significantly from those of the SLE patients. None of the healthy individuals had any form of autoimmune disease.

### 2.2. Laboratory Parameters and Atherosclerosis Risk Factors

Blood samples for laboratory parameter determination were collected from the SLE patients and healthy individuals, always at the same time of day, after at least 8 h of fasting. The assessed variables included VEGF, fibrinogen, total cholesterol, direct LDL and direct HDL cholesterol, direct triglycerides, glucose, homocysteine, uric acid, C-reactive protein (CRP), the erythrocyte sedimentation rate (ESR), and serological markers (described in detail in Section 2.3).

Serum was stored at −80 °C until VEGF measurements using the sensitive ELISA method with the Human VEGF Immunoassay Quantikine kit (R&D Systems, Minneapolis, MN, USA). All analyses and calibrations were performed in duplicate and were read using a BioTek PowerWave XS, BioTek Instruments, Winooski, VT, USA.

The National Cholesterol Educational Program Adult Treatment Panel III criteria were used to identify risk factors for atherosclerosis [34]. We assessed the presence of traditional risk factors for atherosclerosis: lipid metabolism disorders (total cholesterol > 190.0 mg/dL, low-density lipoprotein cholesterol [LDL] > 115.0 mg/dL, high-density lipoprotein [HDL] cholesterol in men < 40.0 mg/dL and in women < 45.0 mg/dL, triglycerides > 150.0 mg/dL), overweight and obesity based on BMI calculations (overweight when the BMI was 25 to <30, obesity when the BMI was ≥30), smoking habits, diabetes, hypertension, oral contraceptive use, and a positive family history of CVD.

Urine testing was performed using Siemens Multistix test strips complemented by urinary sediment evaluations. Proteinuria was estimated from the 24-h urine albumin excretion (g/day). Renal function was assessed on the basis of the plasma creatinine concentration (μmol/L) and the estimated Glomerular Filtration Rate (eGFR), as determined by the MDRD Study equation. When defining kidney involvement, proteinuria of ≥0.5 g/day or an eGFR of <50% was considered abnormal.

### 2.3. Autoantibody Profiles

The antiphospholipid antibodies (aPLs) included anti-cardiolipin antibodies (aCL) of the IgG, IgM, and IgA classes; anti-beta2 glicoprotein I antibodies (aβ2-GPI) of the IgG, IgM, and IgA classes determined through the immunoenzymatic method (EUROIMMUN AG Medizinische Labordiagnstika ELISA tests, Lübeck, Germany); lupus anticoagulant (LA) assessed with coagulometric tests [33]; and anti-prothrombin antibodies (aPT) of the IgG, IgM, and IgA classes (ELISA test, AESKU.LAB DIAGNOSTIKA, Wendelsheim, Germany).

Anti-neutrophil cytoplasmic antibodies (ANCAs) were assessed via indirect immunofluorescence assays (IIFAs) and monospecific ELISA tests to detect anti-proteinase 3, anti-myeloperoxidase, anti-lactoferrin, anti-cathepsin G, anti-elastase, and anti-bactericidal permeability-increasing protein antibodies (EUROIMMUN AG Medizinische Labordiagnostika tests, Germany).

Antinuclear antibodies (ANAs) were assessed on the HEp-2 cell line using IIFAs. Monospecific ELISA analyses were performed to identify anti-double-stranded DNA (adsDNA), anti-Sm, anti-histone, anti-nucleosome, anti-rybosomal P protein, anti-SS-A/Ro, anti-SS-B/La, and anti-U1-RNP antibodies (EUROIMMUN AG Medizinische Labordiagnostika tests, Germany).

Anti-endothelial cell antibodies (AECAs) were determined on human umbilical vein endothelial cells using IIFAs (EUROIMMUN AG Medizinische Labordiagnostika tests, Germany).

Anti-oxidized low-density lipoprotein antibodies (aoxLDL) were analyzed via the ELISA method with synchronous assessments of IgG and IgM classes (IMTEC Immunodiagnostika, Berlin, Germany).

### 2.4. Radiological Examinations

All patients with SLE and all healthy individuals underwent imaging examinations with an HDI 3500 (ATL) and a 5–12 MHz linear transducer by the same radiologist experienced in vascular ultrasound.

Early atherosclerotic lesions were identified as an increase in the carotid intima–media thickness (cIMT). cIMT measurements were performed via B-mode ultrasound according to previously described procedures [35].

The cIMT is highly variable among populations [36,37], so we determined the reference and abnormal ranges of the cIMT based on measurements in the healthy individuals. Values of ≤0.65 mm were considered to be within the normal range; measurements between 0.66 mm and 0.86 mm were considered moderate; and values of >0.86 mm were considered a high cIMT.

Atherosclerotic plaque screening was performed with B-mode ultrasound in the carotid and lower-limb arteries (the tibial; popliteal; superficial, deep, and common femoral; and iliac arteries) [38].

The ankle–brachial index (ABI) was calculated as the ratio of the systolic pressure measured in the posterior tibial and dorsal arteries of both feet to the systolic pressure in the brachial artery. ABI values of <1.0 were considered abnormal [39].

The high resistance index (HRI) was determined through Doppler ultrasonography according to a previously described procedure [40].

The systolic and diastolic functions of the left ventricle were assessed via echocardiography using a Philips EPIC 5G device (Philips Healthcare, Amsterdam, The Netherlands) with an S5-1 broadband sector array transducer according to previously published guidelines [41,42].

### 2.5. Statistical Analysis

The equality of the distributions of continuous variables was checked using the Kolmogorov–Smirnov test. Mann–Whitney and t-Student tests were used to compare the continuous variables. Data are described as the mean ± standard deviation or median (Q1, Q3). The logistic regression model was used for categorical variables. The *r_s_* values of correlations were also determined using Spearman’s rank correlation test. The probability (*p*) was assessed using a chi-square test or Fisher’s exact test. The results are presented as *p*, the odds ratio (OR), and the 95% Confidence Interval (95% CI). Findings were considered statistically significant at *p* < 0.05, while *p* values between 0.05 and 0.1 were considered weak evidence or trends. All statistical analyses were performed with STATISTICA version 11.0, license number 30110532736, StatSoft Inc., Tulsa, OK, USA.

## 3. Results

The demographic, clinical, laboratory, and therapeutic characteristics of the patients and the controls are presented in Table 1, Table 2 and Table 3.

We did not find statistically significant differences between the VEGF serum concentrations for the whole groups of SLE patients and controls (*p* = 0.735). On the basis of the 25th (192.60 pg/mL) and 75th (382.40 pg/mL) percentiles of the VEGF measurements obtained in the control group, the VEGF levels were divided into categories of low (<192.60 pg/mL) and high (>382.40 pg/mL) (Table 4).

When compared to the controls, specific SLE patient subgroups showed significant differences with regard to clinical, serological, and therapeutic characteristics. The mean value (M) of the VEGF concentrations in the controls was 282.3 pg/mL. Significantly higher VEGF levels were found in patients with relaxation disorders (M = 548.6, *p* = 0.014), the presence of atherosclerotic plaque in the iliac artery (M = 412.5, *p* = 0.035), aPT IgA (M = 625.8, *p* = 0.001), and aβ2-GPI IgA (M = 459.4, *p* = 0.021). Higher VEGF serum concentrations in comparison with the controls were also confirmed in patients with the highest values of cIMT (M = 465.1, *p* = 0.065), as well as those with the presence of LA (M = 445.7, *p* = 0.060), aCL IgG (M = 404.6, *p* = 0.090), aPT of all isotypes (M = 403.0, *p* = 0.068), and anti-histone antibodies (M = 406.1, *p* = 0.077), but these results presented only a statistical trend.

SLE activity was illustrated by the SLEDAI score. Low activity was confirmed in a majority of patients (51.8%), moderate activity in 38.6%, and high activity in 9.6%. No statistically significant correlations between the activity index and VEGF concentrations were found. However, there were important associations between clinical and immunological markers of SLE activity and VEGF levels. A significant association was found between low serum VEGF concentrations and a reduced risk of vasculitis (*p* = 0.038), as well as adsDNA coexistence (*p* = 0.034). Lupus patients with lower VEGF levels also presented a trend toward lower ESR values (*p* = 0.080) (Table 5). On the other hand, there was a trend toward a relationship between high VEGF levels and an increased risk of anti-histone antibody occurrence (*p* = 0.83) (Table 6).

Low VEGF levels were also importantly associated with a lower risk of atherosclerotic plaque development in the iliac artery (*p* = 0.049). There was a tendency toward a lower risk of common femoral artery plaque development (*p* = 0.065) in patients with lower VEGF levels. A comparison of SLE patients with and without renal involvement, defined as proteinuria of ≥0.5 g/day or an eGFR of <50%, showed no differences regarding VEGF concentrations; however, in patients with decreased VEGF serum levels, there was a trend toward a reduced risk of lupus nephritis development (*p* = 0.056). Additionally, low VEGF concentrations tended to be associated with decreased cIMT measurements (*p* = 0.098). There were also significant correlations between low VEGF levels and reduced risks of the coexistence of several autoantibodies, such as AECA (*p* = 0.023), aPT IgA (*p* = 0.022), and aβ2-GPI IgA (*p* = 0.015). The associations with decreased risks of other aPLs’ cooccurrence showed weak statistical evidence: aPT IgG/IgM/IgA (*p* = 0.077) and aβ2-GPI IgG/IgM/IgA (*p* = 0.067). Moreover, there was a trend toward an association between VEGF levels of <192.40 pg/mL and a lower risk of aoxLDL coexistence (*p* = 0.056).

Conversely, VEGF concentrations of >382.40 pg/mL were significantly related to a higher risk of development of cardiac abnormalities like relaxation disorders (*p* = 0.011). Moreover, there were significant associations between high VEGF levels and the coexistence of serological markers, including aPT IgA (*p* = 0.004), aβ2-GPI IgA (*p* = 0.032), and aoxLDL (*p* = 0.050). Additionally, patients with high VEGF levels tended to present higher frequencies of other immunological biomarkers, including LA (*p* = 0.059), aCL IgG (*p* = 0.087), and aβ2-GPI IgG/IgM/IgA (*p* = 0.057).

The clinical and immunological characteristics of SLE patients with low and high serum VEGF concentrations are presented in Table 5 and Table 6.

The Spearman’s rank correlation test confirmed significant positive correlations between VEGF serum concentrations and aPT IgA (*p* = 0.002), aβ2-GPI IgA (*p* = 0.021), aβ2-GPI IgG/IgM/IgA (*p* = 0.026), and aoxLDL (*p* = 0.044). There was also weak statistical evidence of positive correlations between VEGF levels and disease duration (*p* = 0.058), cIMT (*p* = 0.052), aPT IgG/IgM/IgA (*p* = 0.078), and AECA (*p* = 0.080). Furthermore, a trend was shown toward a negative correlation between VEGF serum levels and EF (*p* = 0.098). The results are summarized in Table 7.

We also compared SLE patients with comorbidities like diabetes, hypertension, and CVD with SLE patients without these complications (Table 8). Patients with comorbidities were significantly older (*p* = 0.000) and presented higher values of cIMT (*p* = 0.000), total cholesterol (*p* = 0.009), LDL cholesterol (*p* = 0.017), homocysteine (*p* = 0.002), uric acid (*p* = 0.000), and BMI (*p* = 0.030). However, VEGF serum concentrations did not differ between these groups (*p* = 0.327).

No differences were found among SLE groups receiving various treatment regimens in terms of serum VEGF levels (all *p* >0.05). There was only weak statistical evidence of a positive correlation between VEGF concentrations and azathioprine (AZA) use in the management of lupus patients (*p* = 0.064) (Table 7).

## 4. Discussion

The current cross-sectional, original study aimed to evaluate the relationships between VEGF serum levels and atherosclerosis, atherosclerotic risk factors, cardiovascular manifestations, other organ involvement, and autoantibody profiles in SLE patients. The main findings of our research showed significant correlations between low VEGF serum concentrations and reductions in the risk of developing vasculitis and atherosclerotic changes in arteries in the lower extremities. Moreover, the frequencies of selected autoantibodies, including adsDNA, AECAs, and aPLs, were decreased in patients with lower VEGF levels. On the other hand, higher VEGF concentrations were significantly related to an increased risk of relaxation disorder development and the presence of aPLs and aoxLDL in the analyzed SLE cohort.

Our previous studies on the role of angiogenic cytokines in SpAs revealed the involvement of VEGF in the pathogenesis of psoriatic arthritis (PsA) [12] and ankylosing spondylitis [14].

Furthermore, our earlier research on cardiovascular risk in lupus patients confirmed that classical atherosclerotic risk factors alone fail to fully account for accelerated and often premature atherosclerosis, and disease-associated factors like the coexistence of APS, presence of autoantibodies, chronic inflammation, and treatment strategy are of vital importance [43].

Further studies documented the additional role of rare aPLs such as anti-phosphatidylethanolamine (aPE) and anti-phosphatidylserine (aPS) [44], as well as interleukin (IL)-23 [45], in atherothrombosis development in the course of SLE.

All these observations provoked further research on the pathogenesis of vascular involvement in SLE. Below, we present a detailed analysis of the relationships between VEGF serum concentrations and clinical and serological SLE characteristics.

We found no significant difference between VEGF levels in SLE patients and controls. This is contrary to other reports that showed higher VEGF concentrations in lupus patients compared to healthy volunteers, as summarized in a recent meta-analysis [17]; however, the authors stated that, on the basis on 22 reports, the certainty of the evidence was low. Furthermore, in vitro studies showed a significant decrease in VEGF secreted by progenitor endothelial cells and myelomonocytic circulating angiogenic cells in SLE patients compared to controls, which was also confirmed by an assessment of VEGF serum levels [46]. Additionally, the VEGATS study [26] documented statistically higher VEGF levels in SLE patients compared to controls in plasma samples but not in serum. On the other hand, another study did not refer to a control group at all [18] or even showed higher VEGF serum levels in healthy individuals than in SLE patients [47]. Moreover, our previous studies in SpAs patients did not show any significant differences between PsA patients and controls in terms of VEGF serum concentrations, which could be attributed to the low activity of the disease. Similarly, in the current study, more than 50% of the SLE patients presented low SLEDAI scores, which could have resulted in the lack of significant difference regarding VEGF concentrations between the total patient group and the controls. However, the detailed analysis of SLE patient subgroups showed importantly higher VEGF serum levels compared to the controls, particularly in patients with relaxation disorders, atherosclerotic plaque in lower-extremity arteries, and selected aPLs.

Taking into account all the abovementioned aspects, we decided to determine the cut-off value and reference range of serum VEGF concentrations on the basis of measurements in healthy volunteers, establish low and high VEGF levels, and analyze their relationship with disease characteristics, putting particular emphasis on cardiovascular risk factors and atherosclerosis.

A recent meta-analysis underlined VEGF’s dual role in atherosclerosis. VEGF upregulation as a result of a pro-inflammatory and hypoxic state may reflect a compensatory mechanism to maintain structural and functional integrity of the endothelium. However, there is also evidence for a detrimental role in atherosclerosis (suppression of repair mechanisms in endothelial cells, stimulation of monocytes, activation of smooth muscle cells, and consequent initiation of atherosclerotic processes). It may also influence atherosclerotic plaque stability by promoting local neovascularization, resulting in plaque rupture and thrombus formation [17].

In our analysis of VEGF’s relationships with cardiac abnormalities and atherosclerotic indices, we documented a positive correlation with relaxation disorders, as well as a trend toward a positive correlation between VEGF levels and cIMT measurements in the patient group. On the other hand, there was a tendency toward a reverse association with EF values. Moreover, low VEGF concentrations were associated with a decreased risk of atherosclerotic plaque development in lower-extremity arteries. The VEGATS study [26] supported our results by also showing a positive correlation between VEGF levels and cIMT, pointing at VEGF as a potential marker of accelerated atherosclerosis in SLE. The authors underlined that the well-recognized stimuli for VEGF production are inflammation and IL-17, which are directly associated with atherosclerosis development in SLE patients. Our previous study revealed the significant role of IL-23 in lupus pathogenesis and its impact on atherothrombosis development [45]. All these observations indicate the possible role of the IL-17–IL-23 axis in atherosclerosis progression in the course of SLE based on its proinflammatory and proangiogenic properties.

The role of serum VEGF in atherosclerosis and CVD development has also been reported in the general population, showing close associations with atherosclerosis-accelerating factor [48], CVD events [49], and CAD severity [50].

Our study showed a significant association between VEGF serum concentrations and relaxation disorders, as well as a tendency toward an inverse correlation of VEGF levels with EF values in SLE patients. SLE patients with relaxation disorders showed significantly higher VEGF serum levels compared to the controls. Both relaxation disorders and decreased EF are associated with heart failure, and relaxation disorders are the earliest manifestations of left-ventricular diastolic dysfunction in cardiac involvement. It is of note that left-ventricular diastolic disorders might represent isolated heart dysfunction, so early identification of these patients is crucial in their management and further CVD risk stratification. As VEGF plays an important role in heart morphogenesis, myocardial contractility, and wound healing [51], and because elevated levels of VEGF in CVD are usually associated with poor prognosis and disease severity [51], VEGF measurements might be of great importance for identifying patients at risk of CVD and heart failure, including those suffering from SLE.

Moreover, we found a trend toward a correlation between VEGF levels and the duration of SLE. Taking into consideration the cardiovascular risk and bimodal pattern of mortality in SLE patients [52], this finding might be of practical clinical importance in identifying patients at risk, as the second peak of mortality is associated with inactive SLE, long-term treatment with glucocorticosteroids, and a positive history of cardiovascular events. In this regard, VEGF measurements seem to be a useful tool not only in general cardiovascular risk stratification, but especially in assessing the risk of mortality from cardiovascular issues in patients with a long disease duration.

We also confirmed a significant positive correlation between VEGF serum levels (especially those >382.40 pg/mL) and the presence of aoxLDL. Unfortunately, we were not able to distinguish between aoxLDL of the IgG and IgM classes, as the test we used permitted only synchronous measurement of both isotypes.

Antibodies against oxLDL have been explored in terms of cardiovascular risk in the general population as well as in SLE patients, with discrepant results. A review of the role of aoxLDL in the general population, focused on patients with and without established CAD (studies performed in patients with autoimmune diseases were excluded from the analysis), showed that IgM aoxLDL seems to be protective against more severe cardiovascular events. However, the divergent results in the reports indicate that the relation with the IgG isotype is more complicated [53]. The authors hypothesized that IgM aoxLDL has homeostatic properties maintaining the balance of atherosclerosis development. Stimuli such as inflammation or classical factors for CVD can cause immune dysregulation, resulting in immunoglobulin switching class to IgG and accelerating atherosclerotic plaque deposition and rupture [53].

In SLE patients, oxLDL may contribute to arterial disease, and IgG antibodies against oxLDL can be markers of disease distinguishing SLE cases from SLE controls or serve as a marker of SLE-related arterial disease [54]. Furthermore, Lopez et al. [55] confirmed in SLE patients that oxLDL/beta2GPI complexes and IgG aoxLDL/beta2GPI antibodies contribute to the development of autoimmune-mediated atherosclerosis.

However, like in the general population, the exact role of IgG aoxLDL in atherogenesis is unclear. On the one hand, experimental studies on animal models showed that IgG aoxLDL enables foam cell formation through the receptor-mediated uptake of oxLDL by macrophages [56]. On the other hand, studies in humans have suggested that IgG aoxLDL can block such uptake [56]. Thus, there is still a need for further research to fully elucidate this issue.

Correspondingly, little is known about the relationship between VEGF levels and aoxLDL. An experimental in vitro study showed the protective role of VEGF against oxLDL toxicity to endothelia [57]. This effect is mediated by an intracellular glutathione-dependent mechanism via the VEGF receptor KDR/Flk-1. OxLDL has chemotactic, immune-stimulatory, and toxic properties and can induce defects in endothelial cell integrity, resulting in increases in permeability, platelet adhesion, and thrombogenicity. The preventive effect of VEGF may be beneficial in atherogenesis through its maintenance of endothelial morphology and function [58]. In this regard, the higher VEGF levels that we observed in SLE patients in association with aoxLDL might be an effect of the protective role of VEGF in atherogenesis and its release from endothelial cells in response to oxidative stress and oxLDL generation that induces aoxLDL production.

We also confirmed a significant correlation between VEGF levels and aPLs presence in lupus patients. Our earlier studies showed that selected aPLs may contribute to atherosclerosis development in SLE [44,59]. In the current study, we found significant positive correlations between VEGF levels and the presence of aPT and aβ2-GPI. There was also a trend toward association between high VEGF levels and aCL and LA occurrence. SLE patients with selected aPLs present in their blood showed significantly higher VEGF serum concentrations in comparison with the controls. These results may reflect direct endothelial cell activation by aPLs.

Williams et al. [11] confirmed elevated VEGF levels in primary APS patients, especially those with arterial thrombosis. VEGF concentrations were positively correlated with tissue factor (TF) expression, revealing a potential pathomechanism of circulating aPLs that can promote VEGF secretion and causing TF expression and initiating coagulation [11]. Moreover, Cuadrado et al. [60] documented increased expression of VEGF in monocytes from APS patients. The authors concluded that this cytokine may be involved in aPL-mediated monocyte activation and TF expression, contributing to the proinflammatory–prothrombotic phenotype of monocytes in APS patients [60]. These mechanisms may also be important in atherothrombosis development in SLE patients showing close interactions between aPLs and VEGF.

We also confirmed that lower VEGF levels are associated with a decreased risk of AECAs occurrence, which was also reflected by a trend toward positive correlation between VEGF serum levels and the presence of AECAs in lupus patients. Our previous study showed a direct association between AECAs and subclinical atherosclerosis development in SLE [61]. In this regard, the association between VEGF and AECAs may reflect autoantibodies’ direct effects on endothelia, as they can bind to structural antigens and molecules that adhere to endothelial cells, leading to their activation and dysfunction. Indeed, a study by Cieślik et al. [62] showed significantly higher levels of endothelial cell activation markers in SLE patients positive for AECAs. However, they did not analyze VEGF serum concentrations.

We analyzed other clinical characteristics of SLE and their relation to VEGF levels. We did not confirm a significant correlation between VEGF and SLEDAI scores in SLE patients. Many studies have documented close associations between VEGF and disease activity indices [17,21,22,23,24,25]; on the other hand, some have failed to demonstrate such a relationship [63]. One explanation for this discrepancy might be related to the very low number of patients with the highest SLEDAI score in our study. More than 50% of the lupus patients presented low SLEDAI scores. Still, relationships between VEGF levels and several variables associated with SLE activity were proven. We found a significant correlation between low VEGF serum concentrations and a decreased risk of adsDNA presence. Many studies have shown a direct positive correlation between VEGF and adsDNA [18,22,23]. We also found a similar correlation between VEGF levels of <192.60 pg/mL and reduced risk of vasculitis. We indicated trends toward association between lower VEGF concentrations and a reduced risk of lupus nephritis development, as well as decreased ESR values in the analyzed SLE patients. Other studies also found an association between VEGF and ESR values [21,25]. In relation to nephropathy, research results are contradictory. Some confirmed a positive correlation between VEGF and renal involvement in SLE patients [21], while others have not shown such a relationship [63]. Our results indicate that VEGF levels may serve as an SLE activity indicator; in patients with low VEGF concentrations, other parameters related to activity and disease exacerbation were also decreased. Moreover, we revealed an interesting trend toward a correlation between high VEGF levels and the presence of anti-histone antibodies, which may also serve as a marker of disease activity and flare. Thus, VEGF levels may relate to SLE activity, but the immunological background seems to be more complex and is rather attributed to the interplay between VEGF, autoantibodies and inflammatory factors, than to the independent pathogenic VEGF’s role. Further studies are necessary to fully elucidate this issue.

Finally, we found a noteworthy trend toward a positive correlation between VEGF levels and AZA use in lupus patients. The toxic effect of this drug on endothelial cells has been shown in in vitro studies [64] as the endothelium plays an active role in the metabolization of immunosuppressants, including AZA [65]. Furthermore, animal model studies revealed that AZA can induce vascular calcification and an increase in VEGF levels, especially in a long-term treatment scheme [66]. The impact of AZA treatment on cardiovascular risk and the pathophysiology of vessel walls was also confirmed in clinical studies [67,68]. Considering the high atherosclerotic and CVD risks in SLE patients, these observations are very important for patient management in terms of vascular protection and reducing cardiovascular risk.

To summarize, our study demonstrated that one of the pathomechanisms of vascular disorder development in the course of SLE may be a complex interplay between autoantibodies and angiogenic factors with a direct impact on the endothelium. The advantage of this study’s strategy was that both VEGF concentrations—low and high—were taken into account and correlated with SLE characteristics, showing their practical significance: higher levels were related to higher atherosclerotic and CVD risks, while lower levels indicated decreased SLE activity via reduced risks of clinical, immunological, and inflammatory variable coexistence related to disease activity and flare.

However, there are some limitations to our study that need to be mentioned. We analyzed only serum VEGF without any comparison with measurements from plasma samples; it is known that the type of biological material is important for results interpretation [69]. We measured only circulating VEGF without evaluating local VEGF expression; e.g., that in atherosclerotic plaques or renal biopsies. We focused only on VEGF without any analysis of other angiogenic factors. Additionally, there was a small number of patients with high disease activity and clinically overt CVD in our cohort. Only 8 patients presented the highest SLEDAI scores, 4 had a previous history of myocardial infarction, 9 had CAD, 2 had a history of transient ischemic attacks, and 10 had a history of stroke. We compared SLE patients without and with comorbidities like diabetes, hypertension, and CVD manifestations. We found significant differences between the cohorts in terms of cIMT, age, selected lipids, homocysteine and urine acid levels, and BMI. These findings reflected our previous results showing the significant impact of classical atherosclerotic risk factors on CVD development in lupus patients [43]. However, we did not find important differences between the analyzed SLE subcohorts in terms of VEGF concentrations, disease duration, inflammatory markers, activity scores, or other atherosclerosis indices like ABI and HRI, so we decided not to exclude those patients from the analysis. We did not find significant differences in terms of VEGF concentrations between the total group of SLE patients and the controls. However, for specific lupus subgroups, we were able to confirm importantly higher VEGF levels compared to healthy individuals, especially in patients with relaxation disorders, the presence of atherosclerotic plaque in lower-extremity arteries, and the occurrence of selected aPLs, indicating VEGF’s potential role in the development of cardiovascular and atherothrombotic complications, but in combination with other immunological factors, not as an independent pathogenetic factor. The analyzed cohort represented clinical heterogeneity. SLE is known from its heterogeneity by definition, and our cohort reflects the clinical reality; however, clinical diversity may indeed cause bias of study results, hence it is necessary to conduct further studies in more categorized groups of patients in terms of clinical symptoms and disease activity. Furthermore, we analyzed the synchronous presence of aoxLDL IgG/IgM without the possibility of assessing the separate influence of particular isotypes on disease characteristics and the risk of atherosclerosis development in SLE patients. Another limitation was the small number of analyzed SLE patients. Our clinic provides consultations for patients from all over western Poland. Not all those who qualified for the study were able to participate, mainly due to logistical difficulties. Additionally, the control sample size was lower than the patient sample size. However, according to statistical analysis rules, if the study group consists of up to 100 individuals and the desired confidence level for the test is 95%, the size of the control group should correspond to at least 20% of the size of the study cohort, and our research fulfilled this basic requirement. As VEGF serum levels might be influenced by age, gender, time of day when the serum was collected, and smoking habits, we matched all these variables between the SLE patients and the controls. The study was arranged as cross-sectional research. A follow-up of the obtained results is in progress. Similarly, comparative analyses with other disease entities are planned for further studies. So far, the results found in SpAs have been published and are the starting point for research on the significance of VEGF in the pathogenesis of rheumatic diseases.

## 5. Conclusions

In conclusion, VEGF’s pathogenetic role in SLE and SLE-related atherothrombosis is manifested in close correlation with aPLs which may enhance their direct impact on endothelium. High VEGF levels are helpful for identifying cardiovascular risk in patients, while low concentrations indicate lower disease activity, as well as a lower risk of organ involvement.

## Figures and Tables

**Table 1:** Demographic and clinical characteristics of systemic lupus erythematosus patients and controls.

Assessed Parameters	Systemic Lupus Erythematosus Patients	Healthy Controls	*p*
	N = 83	N = 20	
	Mean ± SD	Mean ± SD	
	Median (Q1, Q3)	Median (Q1, Q3)	
Age (years)	44.8 (±13.5)	45.2 (±13.3)	0.451
Gender	F = 74, M = 9	F = 17, M = 3	0.423
Disease duration (years)	8.0 (4.0, 12.5)	-	NA
SLEDAI: low, N (%)	43 (51.8)	-	NA
medium, N (%)	32 (38.6)	-	NA
high, N (%)	8 (9.6)	-	NA
APS, N (%)	29 (34.9)	-	NA
renal involvement, N (%)	23 (27.7%)	-	NA
cardiovascular manifestations:			
CAD, N (%)	9 (10.8)	-	NA
MI, N (%)	4 (4.8)	-	NA
ischemic stroke, N (%)	10 (12.0)	-	NA
TIA, N (%)	2 (2.4)	-	NA
Left ventricular function abnormalities:			
EF, N (%)	24 (28.9)	-	NA
Hypokinesis, N (%)	9 (10.8)	-	NA
Relaxation abnormalities, N (%)	10 (12.0)	-	NA
Raynaud’s phenomenon, N (%)	25 (30.1)	-	NA
vasculitis, N (%)	13 (15.7)	-	NA
Thromboembolic disorders, N (%)	17 (20.5)	-	NA
Hypertension, N (%)	33 (39.8)	1 (5.0)	0.000
BMI	25.4 (±4.8)	24.7 (±3.3)	0.258
Diabetes, N (%)	9 (10.8)	0 (0.0)	0.001
Smoking habits, N (%)	29 (34.9)	10 (50.0)	0.121
Oral contraceptive use, N (%)	4/74 (5.4)	4/17 (23.5)	0.057
Family history of CVD, N (%)	4 (4.8)	4 (20.0)	0.062
cIMT (mm)	0.70 (0.65, 0.80)	0.60 (0.60, 0.68)	0.000
ABI right	1.08 (1.03, 1.16)	1.12 (1.06, 1.24)	0.018
ABI left	1.08 (1.02, 1.16)	1.14 (1.08, 1.21)	0.030
HRI right	0.314 (0.252, 0.390)	0.389 (0.365, 0.434)	0.001
HRI left	0.328 (0.217, 0.394)	0.434 (0.375, 0.469)	0.000
Plaques, N (%):			
carotid arteries total, N (%)	20 (24.1)	-	NA
cca, N (%)	3 (3.6)	-	NA
bulb, N (%)	17 (20.5)	-	NA
ica, N (%)	2 (2.4)	-	NA
lower extremities arteries total, N (%)	23 (27.7)	-	NA
iliaca	7 (8.4)	-	NA
cfa	16 (19.3)	-	NA
dfa	1 (1.2)	-	NA
sfa	8 (9.6)	-	NA
popl	3 (3.6)	-	NA
pta	1 (1.2)	-	NA

F—females; M—males; NA—not applicable; SLEDAI—systemic lupus erythematosus disease activity index; APS—antiphospholipid syndrome; CAD—coronary artery disease; MI—myocardial infarction; TIA—transient ischemic attacks; EF—ejection fraction; BMI—body mass index; CVD—cardiovascular disease; cIMT—carotid intima-media thickness; ABI—ankle-brachial index; HRI—high resistance index; cca—common carotid artery; ica—internal carotid artery; iliaca—iliac artery; cfa—common femoral artery; dfa—deep femoral artery; sfa—superficial femoral artery; popla—popliteal artery; pta—posterior tibial artery.

**Table 2:** Laboratory characteristics of systemic lupus erythematosus patients and controls.

Assessed Parameters	Systemic Lupus Erythematosus Patients	Healthy Controls	*p*
	N = 83	N = 20	
	Mean ± SD	Mean ± SD	
	Median (Q1, Q3)	Median (Q1, Q3)	
VEGF (pg/mL)	259.0 (144.10, 377.80)	228.15 (192.60, 382.40)	0.735
CRP (mg/L)	2.6 (1.2, 6.1)	0.0 (0.0, 1.0)	0.009
ESR (mm/h)	30.5 (12.3, 58.0)	8.0 (2.0, 16.0)	0.000
Fibrinogen (mg/dL)	317.0 (274.0, 376.3)	279.0 (247.0, 338.0)	0.035
Homocysteine (µmol/L)	13.9 (11.0, 18.2)	6.4 (5.2, 8.0)	0.001
Uric acid (mg/dL)	4.6 (3.9, 6.0)	3.9 (3.4, 5.2)	0.082
Total cholesterol (mg/dL)	225.2 ± 62.5	224.0 ± 40.4	0.467
LDL-cholesterol (mg/dL)	135.6 ± 49.2	134.8 ± 34.2	0.472
HDL-cholesterol (mg/dL)	57.7 ± 24.7	61.7 ± 12.2	0.180
Triglycerides (mg/dL)	155.5 ± 94.0	137.5 ± 71.6	0.225
Antinuclear antibodies IgG	73 (88.0)	1 (5.0)	0.000
anti-double stranded DNA IgG	32 (38.6)	0 (0.0)	0.000
anti-nucleosome IgG	28 (33.7)	0 (0.0)	0.000
anti-Sm IgG	4 (4.8)	0 (0.0)	0.039
anti-SS-A/Ro IgG	35 (42.2)	1 (5.0)	0.000
anti-SS-B/La IgG	12 (14.6)	0 (0.0)	0.000
anti-rybosomal P protein IgG	3 (3.6)	0 (0.0)	0.042
anti-histone IgG	17 (20.5)	0 (0.0)	0.000
anti-U1-RNP IgG	18 (21.7)	0 (0.0)	0.000
anti-cardiolipin IgG	31 (37.3)	1 (5.0)	0.000
anti-cardiolipin IgM	18 (21.7)	1 (5.0)	0.008
anti-cardiolipin IgA	22 (26.5)	1 (5.0)	0.012
anti-beta2-glycoprotein I IgG	6 (7.2)	1 (5.0)	0.351
anti-beta2-glycoprotein I IgM	20 (24.1)	0 (0.0)	0.000
anti-beta2-glycoprotein I IgA	21 (25.3)	0 (0.0)	0.000
anti-oxidized low-density lipoprotein IgG/IgM	60 (72.3)	0 (0.0)	0.000
anti-prothrombin IgG	9 (10.8)	1 (5.0)	0.001
anti-prothrombin IgM	11 (13.3)	0 (0.0)	0.000
anti-prothrombin IgA	9 (10.8)	0 (0.0)	0.001
lupus anticoagulant	14 (16.9)	0 (0.0)	0.021
anti-neutrophil cytoplasmic antibodies IgG	34 (41.0)	0 (0.0)	0.000
anti-proteinase 3 IgG	0 (0.0)	0 (0.0)	0.231
anti-myeloperoxidase IgG	7 (8.4)	0 (0.0)	0.004
anti-lactoferrin IgG	10 (12.0)	0 (0.0)	0.001
anti-elastase IgG	7 (8.4)	0 (0.0)	0.004
anti-BPI IgG	1 (1.2)	0 (0.0)	0.160
anti-cathepsin G IgG	11 (13.3)	0 (0.0)	0.000
anti-endothelial cell antibodies IgG	38 (45.8)	2 (10.0)	0.000

VEGF—vascular endothelial growth factor; CRP—C-reactive protein; ESR—erythrocyte sedimentation rate; LDL—low-density lipoprotein; HDL—high-density lipoprotein; BPI—bactericidal/permeability-increasing protein; IgG—immunoglobulin G; IgM—immunoglobulin M; IgA—immunoglobulin A; SD—standard deviation; Q—quartile.

**Table 3:** Immunosuppressive therapy and comorbidities treatment of systemic lupus erythematosus patients and controls.

Assessed Parameters	Systemic Lupus Erythematosus Patients	Healthy Controls	*p*
	N = 83	N = 20	
	Mean ± SD	Mean ± SD	
	Median (Q1, Q3)	Median (Q1, Q3)	
Immunosupprissive treatment:			
GCs N (%)/CD	78 (94.0)/8.65 (11.4, 38.1)	-	NA
Cyclophosphamide N (%)/CD	40 (48.2)/6.40 (4.0, 19.4)	-	NA
Azathioprine N (%)/CD	41 (49.4)/12.90 (6.4, 105.2)	-	NA
Chlorambucil N (%)/CD	3 (3.6)/1.03 (0.0, 0.0)	-	NA
Cyclosporine A N (%)/CD	1 (1.2)/16.50 (0.0, 0.0)	-	NA
Methotrexate N (%)/CD	5 (6.0)/13.58 (0.0, 0.1)	-	NA
Chloroquine N (%)/CD	49 (59.0)/239.60 (59.2, 241.6)	-	NA
Comorbidities treatment:			
Statins, N (%)	22 (26.5)	-	NA
Angiotensin-converting enzyme inhibitors, N (%)	33 (39.8)	1 (5.0)	0.000
Metformin, N (%)	9 (10.8)	-	NA
Acetylsalicylic acid, N (%)	35 (42.2)	-	NA
Antithrombotic treatment, N (%)	8 (9.6)	-	NA

GCs—glucocorticosteroids; CD—cumulative dose in grams; NA—not applicable.

**Table 4:** Vascular endothelial growth factor serum levels in systemic lupus erythematosus patients and controls.

Serum VEGF Levels [pg/mL]	Systemic Lupus Erythematosus Patients N (%)	Controls N (%)	*p*
<192.60	28 (33.7)	5 (25.0)	0.735
192.60–382.40	35 (42.2)	10 (50.0)	
>382.40	20 (24.1)	5 (25.5)	

VEGF—vascular endothelial growth factor.

**Table 5:** A logistic regression model of the OR of low serum levels of vascular endothelial growth factor in systemic lupus erythematosus patients.

Covariates	Serum VEGF < 192.60 pg/mL	
	OR (95%CI)	*p*
Atherosclerotic plaque in iliac arteries	0.24 (0.04–0.99)	0.049
Atherosclerotic plaque in common femoral arteries	0.25 (0.06–1.09)	0.065
Vasculitis	0.17 (0.03–0.91)	0.038
Lupus nephritis	0.31 (0.09–1.03)	0.056
cIMT	0.39 (0.13–1.19)	0.098
AECA	0.30 (0.11–0.85)	0.023
adsDNA	0.31 (0.11–0.91)	0.034
aPT IgA	0.18 (0.05–0.72)	0.022
aβ2-GPI IgA	0.17 (0.04–0.71)	0.015
aPT IgG/IgM/IgA	0.35 (0.11–1.12)	0.077
aβ2-GPI IgG/IgM/IgA	0.17 (0.04–0.71)	0.015
aoxLDL	0.36 (0.13–1.03)	0.056
ESR	0.42 (0.16–1.11)	0.080

VEGF—vascular endothelial growth factor, OR—odds ratio, 95%CI—95% confidence interval, cIMT—carotid intima-media thickness, AECA—anti-endothelial cell antibodies, adsDNA—anti-double stranded DNA antibodies, aPTA—anti-prothrombin antibodies, aβ2-GPI—anti-beta 2 glycoprotein I antibodies, aoxLDL—anti-oxidized low-density lipoprotein antibodies, ESR—erythrocyte sedimentation rate.

**Table 6:** A logistic regression model of the OR of high serum levels of vascular endothelial growth factor in systemic lupus erythematosus patients.

Covariates	Serum VEGF > 382.40 pg/mL	
	OR (95%CI)	*p*
Relaxation disorders	7.96 (1.60–39.46)	0.011
aPT IgA	10.65 (2.12–53.44)	0.004
aβ2-GPI IgA	34.46 (1.11–10.79)	0.032
aoxLDL	4.77 (1.00–22.77)	0.050
LA	4.41 (0.96–12.15)	0.059
aCL IgG	2.53 (0.87–7.34)	0.087
aβ2-GPI IgG/IgM/IgA	2.76 (0.97–7.84)	0.057
anti-histone antibodies	3.11 (0.86–11.22)	0.083

VEGF—vascular endothelial growth factor, OR—odds ratio, 95%CI—95% confidence interval, aPT—anti-prothrombin antibodies, aβ2-GPI—anti-beta 2 glycoprotein I antibodies, aoxLDL—anti-oxidized low-density lipoprotein antibodies, LA—lupus anticoagulant, aCL—anti-cardiolipin antibodies.

**Table 7:** Spearman’s rank correlations between vascular endothelial growth factor serum levels and clinical and serological covariates in systemic lupus erythematosus patients.

Covariates	VEGF concentration	
	*r_s_*	*p*
Disease duration	0.21	0.058
cIMT	0.21	0.052
EF	−0.20	0.098
Azathioprine use	0.21	0.064
aPT IgA	0.33	0.002
aβ2-GPI IgG/IgM/IgA	0.25	0.021
aPT IgG/IgM/IgA	0.20	0.078
aoxLDL	0.22	0.044
AECA	0.19	0.080

VEGF—vascular endothelial growth factor, *r_s_*—Spearman’ correlation coefficient, cIMT—carotid intima-media thickness, EF—ejection fraction, aPT—anti-prothrombin antibodies, aβ2-GPI—anti-beta 2 glycoprotein I antibodies, aoxLDL—anti-oxidized low-density lipoprotein antibodies, AECA—anti-endothelial cell antibodies.

**Table 8:** Comparison of systemic lupus erythematosus patients with coexisting diabetes, hypertension, or cardiovascular disease with patients without comorbidities.

Assessed Parameters	Systemic Lupus Erythematosus Patients Without Comorbidities	Systemic Lupus Erythematosus Patients with Comorbidities	*p*
	N = 42	N = 41	
	Mean ± SD	Mean ± SD	
	Median (Q1, Q3)	Median (Q1, Q3)	
Age, years	40.0 ± 11.0	49.5 ± 11.9	0.000
Duration of the disease, years	8.9 ± 5.3	10.3 ± 7.7	0.165
SLEDAI: low, N(%)	22 (52.4)	21 (51.2)	
medium, N(%)	15 (35.7)	17 (41.5)	0.174
high, N(%)	5 (9.8)	3 (7.3)	
cIMT (mm)	0.68 (0.55, 0.95)	0.75 (0.60, 1.05)	0.000
ABI right	1.10 (0.95, 1.30)	1.07 (0.87, 1.47)	0.406
ABI left	1.09 (0.94, 1.31)	1.08 (0.68, 1.50)	0.369
HRI right	0.314 (0.000, 0.545)	0.318 (0.118, 0.485)	0.399
HRI left	0.331 (0.000, 0.501)	0.316 (0.123, 0.436)	0.489
VEGF (pg/mL)	260.6 (31.2, 1573.5)	262.2 (50.6, 1668.3)	0.327
Total cholesterol (mg/dL)	205.9 ± 53.0	238.9 ± 66.7	0.009
LDL-cholesterol (mg/dL)	122.1 ± 40.5	144.9 ± 52.2	0.017
HDL-cholesterol (mg/dL)	56.0 ± 18.7	58.3 ± 17.3	0.286
Triglycerides (mg/dL)	152.7 ± 131.0	158.5 ± 65.8	0.401
BMI	24.4 ± 4.72	26.5 ± 5.15	0.030
Uric acid (mg/dL)	4.3 (2.0, 7.1)	5.1 (0.0, 10.2)	0.000
Homocysteine (µmol/L)	12.0 (7.5, 25.2)	15.0 (9.1, 40.9)	0.002
ESR (mm/h)	39 (2, 160)	30 (2, 158)	0.496
CRP (mg/L)	0.0 (0.0, 6.9)	0.0 (0.0, 1.0)	0.273

SLEDAI—systemic lupus erythematosus disease activity index; cIMT—carotid intima-media thickness; ABI—ankle-brachial index; HRI—high-resistance index; VEGF—vascular endothelial growth factor; LDL—low-density lipoprotein; HDL—high-density lipoprotein; BMI—body mass index; ESR—erythrocyte sedimentation rate; CRP—C-reactive protein.

## Data Availability

The data are available upon request.

## Abbreviations

The following abbreviations are used in this manuscript:

SLE	Systemic lupus erythematosus
CVD	Cardiovascular disease
VEGF	Vascular endothelial growth factor
APS	Antiphospholipid syndrome
SpA	Spondyloarthropathies
SLICC	Systemic Lupus International Collaborating Clinics
SLEDAI	Systemic Lupus Erythematosus Activity Index
LDL	Low-density lipoprotein cholesterol
HDL	High density lipoprotein cholesterol
BMI	Body mass index
CRP	C-reactive protein
ESR	Erythrocyte sedimentation rate
eGFR	Estimated Glomerular Filtration Rate
MDRD	Modification of Diet in Renal Disease
aPLs	Antiphospholipid antibodies
aCL	Anti-cardiolipin antibodies
aβ2-GPI	Anti-beta2 glicoprotein I antibodies
LA	Lupus anticoagulant
aPT	aAti-prothrombin antibodies
ANCA	Anti-neutrophil cytoplasmic antibodies
ANA	Antinuclear antibodies
adsDNA	Anti-double stranded DNA antibodies
AECA	Anti-endothelial cell antibodies
aoxLDL	Anti-oxidized low-density lipoprotein antibodies
cIMT	Carotid intima-media thickness
ABI	Ankle-brachial index
HRI	High resistance index
OR	Odds ratio
CI	Confidence Interval
EF	Ejection fraction
M	Mean
PsA	Psoriatic arthritis
IL	Interleukin
AZA	Azathioprine
